# Examining the role of the temporo-parietal network in memory, imagery, and viewpoint transformations

**DOI:** 10.3389/fnhum.2014.00709

**Published:** 2014-09-16

**Authors:** Kiret Dhindsa, Vladislav Drobinin, John King, Geoffrey B. Hall, Neil Burgess, Suzanna Becker

**Affiliations:** ^1^School of Computational Science and Engineering, McMaster UniversityHamilton, ON, Canada; ^2^Neurotechnology and Neuroplasticity Lab, Department of Psychology Neuroscience and Behaviour, McMaster UniversityHamilton, ON, Canada; ^3^Psychology and Language Sciences, University College LondonLondon, UK; ^4^Institute of Cognitive Neuroscience, University College LondonLondon, UK

**Keywords:** spatial cognition, navigation, learning, fMRI, hippocampus

## Abstract

The traditional view of the medial temporal lobe (MTL) focuses on its role in episodic memory. However, some of the underlying functions of the MTL can be ascertained from its wider role in supporting spatial cognition in concert with parietal and prefrontal regions. The MTL is strongly implicated in the formation of enduring allocentric representations (e.g., O'Keefe, 1976; King et al., 2002; Ekstrom et al., 2003). According to our BBB model (Byrne et al., 2007), these representations must interact with head-centered and body-centered representations in posterior parietal cortex via a transformation circuit involving retrosplenial areas. Egocentric sensory representations in parietal areas can then cue the recall of allocentric spatial representations in long-term memory and, conversely, the products of retrieval in MTL can generate mental imagery within a parietal “window.” Such imagery is necessarily egocentric and forms part of visuospatial working memory, in which it can be manipulated for the purpose of planning/imagining the future. Recent fMRI evidence (Lambrey et al., 2012; Zhang et al., 2012) supports the BBB model. To further test the model, we had participants learn the locations of objects in a virtual scene and tested their spatial memory under conditions that impose varying demands on the transformation circuit. We analyzed how brain activity correlated with accuracy in judging the direction of an object (1) from visuospatial working memory (we assume transient working memory due to the order of tasks and the absence of change in viewpoint, but long-term memory retrieval is also possible), (2) after a rotation of viewpoint, or (3) after a rotation and translation of viewpoint (judgment of relative direction). We found performance-related activity in both tasks requiring viewpoint rotation (ROT and JRD, i.e., conditions 2 and 3) in the core medial temporal to medial parietal circuit identified by the BBB model. These results are consistent with the predictions of the BBB model, and shed further light on the neural mechanisms underlying spatial memory, mental imagery and viewpoint transformations.

## 1. Introduction

The precise role of the hippocampus in memory has been the subject of much debate. A large body of evidence points toward a crucial role for this structure in the formation of allocentric spatial representations, based on rodent and non-human primate hippocampal place cell recordings, as well as studies of humans with hippocampal lesions and implanted electrode hippocampal recordings (e.g., O'Keefe, 1976; King et al., 2002; Ekstrom et al., 2003). However, evidence also points toward allocentric representations outside of the hippocampus. For example, neuroimaging of healthy individuals and studies of individuals with lesions implicate the retrosplenial and parahippocampal cortices in memory for scenes and landmarks, navigation to goals and memory across changes of viewpoint (Bohbot et al., 1998; Epstein and Kanwisher, 1998; Aguirre and D'Esposito, 1999; Maguire, 2001; Lambrey et al., 2012; Zhang et al., 2012; Sherrill et al., 2013; Sulpizio et al., 2013).

Considering that information arrives at the sensory receptors in an egocentric frame of reference, e.g., retinocentric in the case of visual input, a transformation must be carried out to translate from egocentric to allocentric co-ordinates. Such a transformation of co-ordinates is a non-trivial calculation for a neural circuit. It is therefore likely that a hierarchy of multiple brain regions is involved in carrying out this transformation, with a gradual emergence of progressively more global, allocentric representations; this is a key assumption underlying the Byrne, Becker, and Burgess (BBB) model of spatial memory (Byrne et al., 2007). Moreover, the BBB model suggests a role for hippocampal neurons in learning conjunctions of allocentric boundary and landmark features, as well as other non-spatial features, explaining the emergence of context-modulated place cells (Anderson and Jeffery, 2003). The BBB model thus sheds light on two major unresolved issues in the literature concerning the role of the hippocampus in memory: (1) What is the role of the hippocampus vs. extra-hippocampal structures in allocentric coding? (2) What is the role of the hippocampus in conjunctive/episodic (including non-spatial) encoding?

Central to the BBB model is an egocentric parietal window that maintains representations of objects, landmarks and boundaries. The parietal window is postulated to be located in the precuneus/medial parietal cortex. The contents of the parietal window can be maintained in working memory through reciprocal fronto-parietal connections. Additionally, object/landmark locations within the parietal window can be continuously updated during real or imagined self-movement through reciprocal connections with the medial temporal lobe. These head-centered and body-centered representations formed in posterior parietal cortex are mapped, via a transformation circuit, into allocentric spatial representations in the parahippocampal region and hippocampus. An egocentric parietal window thus allows one to integrate sensory inputs into an egocentric map, cueing the recall of spatial representations in long-term memory. Conversely, reciprocal connections from the hippocampus to posterior parietal regions allow the products of memory retrieval to generate mental imagery within the parietal window which can be manipulated for the purpose of planning ahead and imagining the future. Other non-spatial contextual features are also integrated at the level of the hippocampus, giving rise to configural memories for places and events.

The BBB model makes several empirical predictions. The first step in mapping from egocentric to allocentric representations involves combining head-centered object representations maintained in the parietal window with allocentric head-direction signals; retrosplenial cortex is anatomically well situated to carry out this computation, as it is reciprocally connected with parietal and medial temporal regions, and receives inputs from areas known to carry head-direction information (Wyss and Groen, 1992; Maguire, 2001; Kobayashi and Amaral, 2003). Thus, we predict that retrosplenial cortex would be engaged whenever egocentric-to-allocentric mappings (or the reverse) are required. Egocentric object/boundary representations modulated by allocentric head direction are in turn transformed into a map of allocentric representations of individual boundaries, objects and landmarks in the parahippocampal cortex. Finally, these object and boundary features are combined at the level of the hippocampus into allocentric representations of particular places (place cells). Thus, according to the BBB model, allocentric coding emerges in at least three levels of representation: in the retrosplenial cortex, in the parahippocampal cortex and in the hippocampus. Whether a given allocentric task requires the hippocampus should depend on whether a conjunction of object/boundary locations is required to solve the task. Thus, orienting to a single landmark might engage the retrosplenial and parahippocampal cortices but may not require the hippocampus. On the other hand, locating an object relative to a configuration of landmarks and other contextual features, thereby uniquely placing it in space and context, should be hippocampal-dependent.

Recent evidence from fMRI studies supports some of the predictions of the BBB model. When participants performed a change detection task while viewing object configurations, trials involving imagined changes in viewpoint (involving both a translation and rotation) were associated with activation of the precucneus, parieto-occipital sulcus/retrosplenial cortex and hippocampus (Lambrey et al., 2012). Similarly, performance of a judgment of relative direction (JRD) task activated the parahippocampal and retrosplenial cortices to a greater degree after learning routes through a virtual town relative to a map-learning condition (Zhang et al., 2012). To further test predictions of this model empirically, we investigated spatial memory retrieval under conditions that impose varying demands on the transformation circuit.

We employed a virtual reality implementation of the JRD task with several conditions, each providing less context and placing a progressively greater burden on memory, mental imagery, and viewpoint transformation: no viewpoint change (REF), a pure rotation of viewpoint (ROT), and (as in Lambrey et al., 2012; Zhang et al., 2012) combined translation and rotation (JRD). A “baseline” condition involving no viewpoint change but including the background scenery and visual feedback was also included for comparison. After learning a configuration of object locations in a virtual environment with easily distinguished distal landmarks, participants underwent fMRI scanning while performing spatial memory and imagery test trials. On each test trial the participant was asked point to an object from either a familiar or novel viewpoint. During REF trials participants were asked to imagine their position and viewpoint were identical to the familiar reference viewpoint they had learned previously before pointing to the cued object. During ROT trials, participants were asked to imagine their position being identical to the position in REF, but that they were instead facing one of the objects and asked to point to a second object. During JRD trials, commonly referred to as a judgment of relative direction (Shelton and McNamara, 1997), participants were asked to imagine they were standing in the position of object X, facing object Y, and then to point to a third object Z.

Thus, in ROT and JRD, participants were asked to imagine a configuration of objects from a changed perspective, which should require the egocentric to allocentric transformation circuit. We analyzed the brain areas which correlated with the execution of these different tasks, and how brain activity in key regions of interest correlated with accuracy in judging the direction of an object after a perspective shift. We hypothesized that all three conditions would require visuo-spatial imagery and therefore activate the parietal window/precuneus, but only ROT and JRD would activate the transformation circuit (retrosplenial cortex) and allocentric representation of objects (parahippocampal cortex) and object configurations (hippocampus). We further predicted that JRD would most strongly activate this circuit, since it requires the most complex transformation (involving both the transformation required in ROT as well as a translation).

## 2. Materials and methods

### 2.1. Ethics statement

The study was approved by the ethics review boards at McMaster University and St. Joseph's Healthcare Hamilton. All participants gave written consent to participate in the behavioral selection experiment, written notice of interest to be considered for scanning, and additional written consent to take part in the fMRI scan on the day of scanning.

### 2.2. Participants

Fifteen participants were included in the final analysis after six were rejected due to excess motion in the scanner and technical issues with scanning, and one elected to withdraw from the study during scanning. All participants were male right-handed McMaster University students (14 undergraduate, 1 graduate) with normal or corrected vision and were classified as gamers (minimum of 10 h a week playing video games). Gamers were chosen due to their experience with operating and navigating in virtual environments, and males were preferred to avoid sex differences in spatial cognition and navigation (Voyer et al., 1995; Parsons, 2004; Levin et al., 2005).

### 2.3. Selection experiment vs. scanning experiment

Participants performed the experiment twice, first outside of the scanner (the “selection experiment”) and second, within the scanner 3–5 weeks later. The initial selection experiment, used to select participants for the scanning session, was run in a quiet testing room on a Lenovo Thinkpad E430 laptop with a 14″ 1366 × 768 resolution display.

During the selection experiment, each participant performed three rounds, each involving a block of each task. A single round included five consecutive blocks of Collect and Replace for learning, followed by one block each of VIS, INVIS, REF, ROT, and JRD, in order (these tasks are defined in detail below). Final JRD accuracy was used to determine whether a participant was invited for a follow-up scanning session so as to select only those who could learn the arena map and infer object-to-object relationsihps well. Participants were required to have either an average JRD error less than one standard deviation below the mean on the final round of the experiment, or average JRD errors less that one standard deviation below the mean on each of the first two rounds of the experiment.

The scanning experiment was conducted at St. Joseph's Hospital in Hamilton, Ontario, Canada. Prior to scanning, each participant performed two complete rounds outside the scanner as done in the selection experiment to refresh their memory of the arena map and instructions for each task. Only the last four pointing tasks (INVIS, REF, ROT, and JRD) were performed in the scanner.

### 2.4. Experimental stimuli and tasks

**Figure 1:**
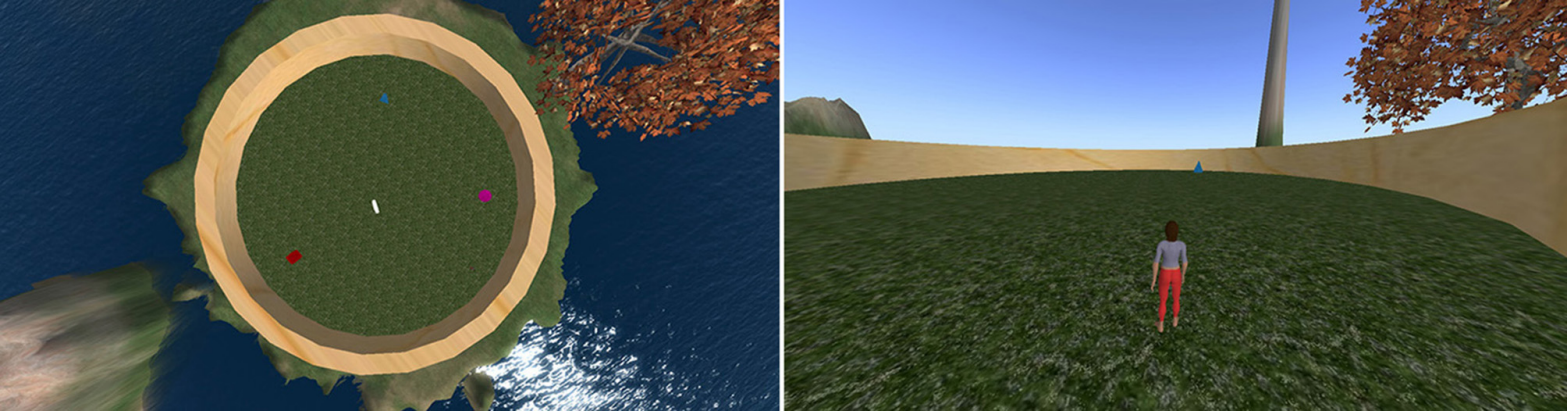
**Left:** Bird's-eye-view of the layout of the environment used for all experiments (avatar not shown). Contains a red cube, a blue pyramid, a white taurus, and a pink sphere. **Right:** View of arena during navigation (only one object visible at a time).

A virtual environment was built in the open-source simulation platform OpenSimulator (Overte Foundation, 2007). All of the tasks took place in a circular arena on a flat grassy ground with visible distal landmarks distributed in the background (see Figure 1). These landmarks included two uniquely shaped hill formations, the sun in a fixed location, and a tree. The environment involved no variation in weather, brightness, or atmosphere.

The circular arena contained four distinct objects set in a consistent spatial configuration used for all tasks and participants. Object locations were chosen so they did not directly align with distal landmarks to encourage participants to encode each object relative to the configuration of landmarks. They were also set so they did not form the vertices of a simple polygon to discourage participants from learning object-to-object spatial relations. Finally, they were set so that all objects were within the field of view from the reference viewpoint used in learning and REF.

#### 2.4.1. Experiment overview

The participant was required to perform several different tasks in this environment. Verbal instructions were given by the experimenter between tasks during the selection experiment. The selection experiment with verbal instructions was repeated immediately before the scanning session to refresh the participant's memory of the object locations and VR controls. No feedback was given for REF, ROT, or JRD, so object-to-object relations could not be learned directly.

All input from the participants involved either navigation using directional keys within the arena (in the selection experiment only) or pointing to objects using an arrow. When navigating (only during the task Collect and Replace, described below), participants controlled an avatar (a virtual character) from a third-person view using the keyboard's directional arrows. When pointing, the participants rotated an arrow, presented in the fronto-parallel plane, to identify the direction of the cued object. In the scanner, participants made pointing responses using buttons on a gamepad-like device that was safe for operation within the scanner. The pointing arrow was rotated clockwise or counter-clockwise in the fronto-parallel plane using two buttons, and a response was made with the third button. On all pointing trials, the initial direction of the arrow was randomized to avoid the interference of proprioceptive memory over visuospatial memory.

**Figure 2:**
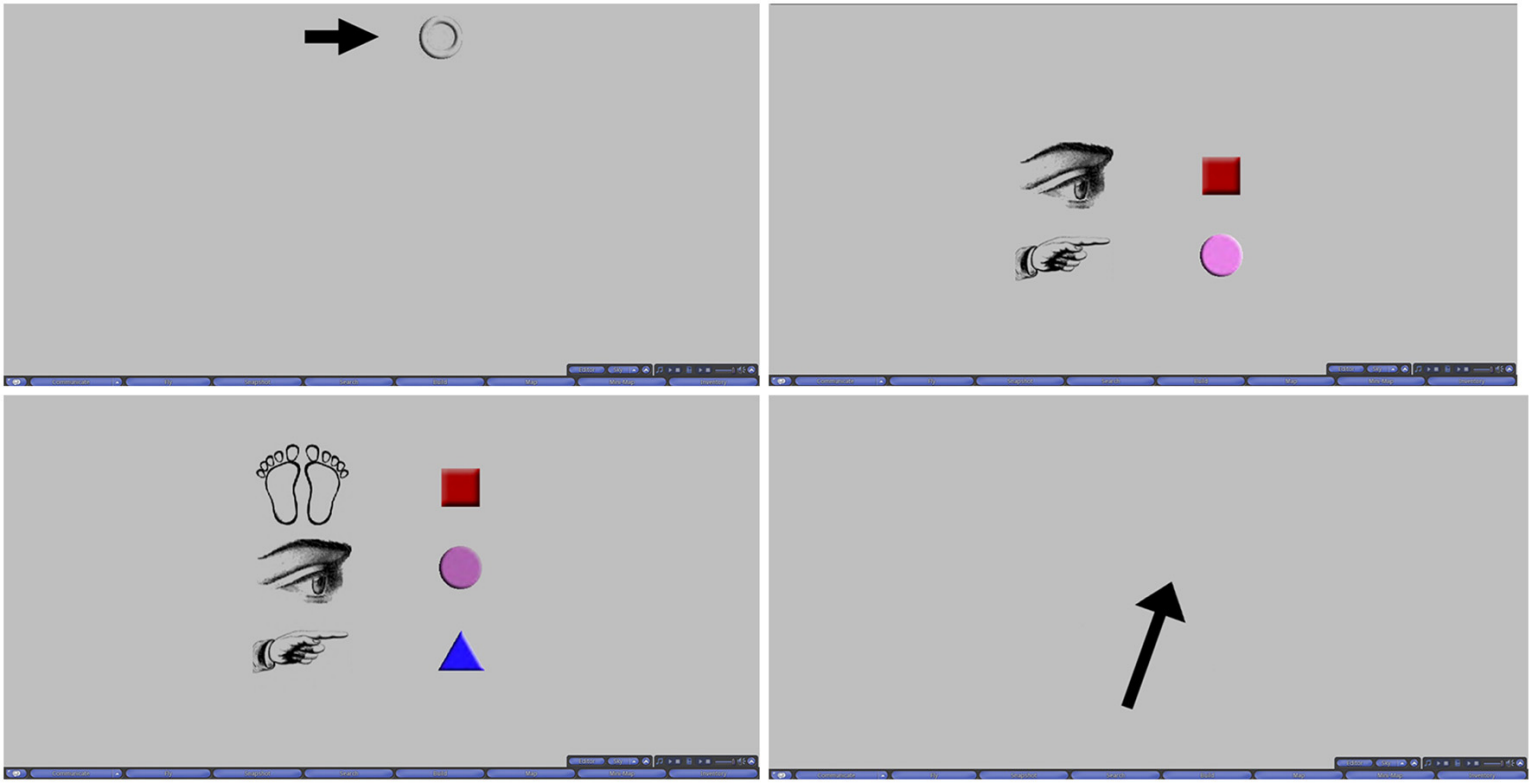
**Top Left:** Cue for VIS, INVIS, and REF. **Top Right:** Cue for ROT. **Bottom Left:** Cue for JRD. **Bottom Right:** Pointing response arrow.

During the scanning portion of the experiment, participants were cued using pictorial instructions in a heads-up display to inform them of the task that was starting and the goal of each trial. Prior to each trial onset, a blank screen was displayed for 3 s. Afterwards, the cue was overlaid on the blank screen for another 3 s (6 and 9 s respectively for the ROT and JRD tasks, whose cues are described more fully below), followed by another blank screen for 3 s. Following this second blank gray screen, the pointing response arrow appeared on the blank screen to indicate that a response could be made. Other than the differences in the cues visually and temporally, all trials across all tasks were presented identically. Cues and the arrow used for pointing response are presented in Figure 2 for each condition.

Participants performed the tasks at their own pace, though trials which lasted longer than 25 s were rejected, as it was deemed likely that the participant had not been sufficiently engaged during the trial. Since each trial required the participant to imagine and reason about the spatial relationships of the objects in the arena before making a response, no strict response time could be imposed. Therefore, response time varied from trial to trial, as evident in Table 1. Given this necessity for self-paced trials, and the strict time limit on individual scanning sessions imposed by the institution housing the fMRI scanner (allowing us 21 min and 48 s per participant), we allowed each participant to complete as many trials as possible within their allotted time. Participants were not given any indication of a time limit or any suggestion that they should perform the trials quickly as to avoid rushing them. The minimum number of rounds completed was 2, and the maximum was 4, with a mean of 2.73 fully completely rounds.

**Table 1:** **Means (standard error of means) of pointing errors and response times**.

	**JRD**	**ROT**	**REF**	**INVIS**
Absolute pointing error (°)	25.56 (4.97)	38.91 (2.64)	6.00 (0.75)	5.27 (0.66)
Response time (s)	15.84 (1.63)	16.47 (1.81)	11.13 (1.08)	12.02 (0.93)

Each round contained a block each of REF, ROT, and JRD (the first two rounds included a block of the baseline task INVIS to orient the participant within the scanner and to check the validity of their responses) with four randomly generated trials in each block. In REF, each object was pointed to once in each block in a random order, but in ROT and JRD, trials were completely randomly generated, i.e., the viewpoint direction and/or position was resampled on each trial. Each round ended with a screen indicating the end of the round, and participants were able to start the next round at their own pace by pressing a button on their input device. On average, 13.6 REF trials, 12.0 ROT trials, and 10.8 JRD trials were completed within the 25 s rejection threshold by each participant.

For each round of the experiment, REF, ROT and JRD were performed in the same order. It was required that REF come first in order to re-establish the reference viewpoint for the ROT condition, which must follow REF in order make use of this freshly re-established viewpoint since ROT uses the same position, albeit a different heading direction. Since the JRD breaks away from this reference viewpoint, it may interfere with performance of the ROT trials if it were to be interposed between REF and ROT. Therefore, there was no counterbalancing of the task order in this experiment. The order during the selection experiment was Collect and Replace, VIS, INVIS, REF, ROT, and JRD, while the order for the scanning eperiment was INVIS, REF, ROT, and JRD.

#### 2.4.2. Collect and replace

Participants performed five rounds of collecting the objects and returning them to their original locations in order to learn the layout of the arena and distal landmarks. Participants were cued to collect each object in the arena one by one in a random order and each object was collected simply by walking to the object. Participants were instructed that they would need to replace each object during the next task and that no visual information aside from the distal landmarks would be avialable during that time, implying that these landmarks should be carefully observed during the collect phase. Only the cued object was visible during each collect trial so that participants were less able to encode the spatial relationships between the objects themselves and were needed to rely on the distal landmarks. All other potential cues were minimized by ensuring the shape and textures of the ground, arena, and sky were consistent throughout.

After each object had been collected, the avatar was teleported to a random location within the arena with a viewpoint in a random direction. With none of the objects visible, the participant was next required to walk close to the cued object's original location in order to replace it. The object appeared for 1 s when the avatar was close enough to the location (within two virtual meters to the object center, which is roughly the height of the avatar) to provide feedback for learning, and disappeared again before the next object was cued. After all of the objects were replaced, the avatar was randomly teleported and the collect phase repeated. After the fifth collect and replace round was completed, the next task was initiated.

#### 2.4.3. Pointing while visible (VIS) and pointing while invisible (INVIS)

Pointing While Visible (VIS) was a calibration task that simply asked the participant to point to each successively cued object using the black pointing arrow (here on a circular white background superimposed on the avatar to avoid the possibility of avatar acting as an additional directional cue). This served the purpose of allowing the participant to establish a familiar viewpoint and to become accustomed to the pointing controls. It also provided an indication of the baseline pointing error, in degrees, for that participant. These errors were checked to ensure that the participant correctly performed the task and that performance was higher on this task than on any of the other more challenging pointing tasks.

Pointing while invisible (INVIS) was identical to pointing while visible except that the objects were not visible (all other aspects of the scene remained visible). The participant was required to point to the location of each object from memory and was provided feedback after each response by the brief reappearance of the target object. Note that this reference viewpoint, used for VIS, INVIS, and REF, had all objects in the field of view, so visual feedback was always possible.

During scanning, VIS was only used as a means to orient the participant to viewing the screen and operating the controls within the scanner, and to check that they were still able to perform the trials with similar accuracy as outside of the scanner. INVIS was used in a similar way, but also for comparison to REF, since the task is similar but without as much reliance on mental imagery. For the remainder of the paper, INVIS will generally be referred to as the baseline task.

#### 2.4.4. Pointing from imagery (REF)

Pointing from imagery required the participant to point to the objects from the same viewpoint as in the previous pointing tasks (the *reference viewpoint*). However, the entire virtual environment was now occluded and only the pointing arrow and cues were visible. Pointing necessarily took place purely from memory without the assistance of distal landmarks and was done with a black arrow on a gray background occluding the entire arena (the same black arrow over a gray background was used for the ROT and JRD tasks as well).

#### 2.4.5. Pointing with rotation (ROT)

Pointing with Rotation differed from the previous pointing tasks in that the participant was instructed to imagine that their point of view was rotated from the reference view established during the previous pointing tasks to a view centered on one of the objects. The environment remained occluded, as in REF, and task structure was identical except for the extra 3 s given to interpret the cue. The cue was changed to reflect the need to illustrate two instructions (Figure 2): which object the participant should center their viewpoint on, and which object they should point to from that viewpoint. As in REF, pointing responses were made with a black arrow on a gray background.

On the first ROT trial of each round, the cue was displayed for 9 s to give the participant extra time to process the cue if they were not sufficiently prepared. On subsequent trials, the cue was presented for only 6 s (3 s for each instruction). As seen in Figure 2, all instructions of the cue were displayed together (similarly for JRD trials). This additional time was especially important (as well as the inclusion of this period in analysis of the fMRI data), because pilot studies and participant surveys both found that many participants engaged in adjusting their imagined viewpoint in steps as they read each instruction of the cue.

#### 2.4.6. Judgment of relative direction (JRD)

The final task was a judgment of relative direction (JRD). This was almost identical to ROT except that a translation was added. Participants were required to imagine standing at the location of one object, facing a second object, and then to point to a third object. A third line of images was added to the cue to illustrate all three components of the instructions (Figure 2). This cue was presented for 9 s. On the first JRD trial of each round, the cue was displayed for 12 s for the same reasons given above in ROT.

### 2.5. fMRI data analysis

Scans were performed with a 3 Tesla General Electric fMRI scanner. A T1-weighted anatomical scan in the axial orientation was obtained prior to functional imaging. The scanning parameters for the anatomical image series were: 3D SPGR pulse; fast IRP sequence; prep time = 450; flip angle = 12; FOV = 240 mm; *TE = 2.2* ms; *TR* = 7.7 ms; 80 slices; slice thickness = 2 mm, no skip.

Functional images were were collected in interleaved axial slices with a GRE-EPI pulse sequence. The field of view was 21 cm with a slice thickness of 2.9 mm and a slice gap of 0.1 mm There were 40 slices per volume with a *TR* of 2600 ms, totalling 500 volumes and a functional scan time of 21 min and 48 s. The *TE* was 25 ms and the flip angle was 90°.

Image processing and statistical analysis were performed using BrainVoyager QX 2.6 (Brain Innovation, Maastricht, The Netherlands) (Formisano et al., 2006; Goebel et al., 2006). Anatomical data were remapped to an iso-voxel size of 1.0 × 1.0 × 1.0 mm with a cubic spline interpolation and a framing cube dimension of 256 points. Each data set underwent manual anterior commissure to posterior commissure alignment. The anatomical 3D data sets were then normalized to Talairach space using linear affine transformation.

The functional data sets were slice-time corrected, 3D motion corrected and realigned to the fifth volume in the series, high-pass filtered at 2 sines/cosines, and normalized to Talairach space (Talairach and Tournoux, 1988). Funtional data series with motion greater than the fMRI voxel size were discarded from analysis as recommended by Formisano et al. (2006). The functional data were then co-registered with the 3D anatomical data allowing for the creation of a 3D aligned time course. The 3D aligned time course data was smoothed with a 6 mm full-width at half-maximum (FWHM) Gaussian filter. Finally, the functional data was masked to filter out noise in the data that fell outside of brain tissue.

A general linear model (GLM) was used to model each participant's data individually. Due to the self-paced nature of each trial and the various possible strategies participants may have used for producing their responses (we found in a survey that most performed the necessary mental imagery during both the cue phase and response phase of the trial), we used the time window from cue onset to response input to measure brain activation. For the ROT and JRD conditions, a parametric model was built by using the standardized pointing errors as an additional regressor. This weighted the brain activity by trial accuracy (i.e., coefficients are found for performance/accuracy regressor). The unweighted brain activity was subtracted from the performance-weighted activity (the z-scores of all regressors needed to be used here so that the regressors were similarly scaled prior to subtraction) to find performance-related activations and to reduce the loss of information when averaging across strategy differences, individual skill differences, and trial-by-trial changes in attention, effort, strategy, and performance. This included the added benefit of filtering brain activations that may have been a result of superfluous processing not contributing to task performance, including processing on-screen visuals. Data from all participants were combined in a random-effects GLM using a participant-averaged mask and an averaged anatomical.

#### 2.5.1. Correction for multiple comparisons

Where possible, we used the false discovery rate correction (FDR) of *p_FDR_* < 0.05, which is usually thought of as a stricter correction than the alternatives when activation is sparse, and less conservative if activated areas are large (Genovese et al., 2002). When the FDR correction was either too strict or too lenient for the specific analysis being run, we used a threshold corrected for family-wise error (FWE) of *p_FWE_* < 0.05, which is another standard. This was done by using Monte Carlo simulations to find the minimum cluster size needed to achieve significance threshold based on an uncorrected per voxel threshold of *p* < 0.005 (Forman et al., 1995). The type of correction and the required minimum cluster size required for each contrast is given in the tables of results. We found the same, and often additional, areas of activation using both methods, but the statistically significant areas were either extremely large or extremely small when using either only FDR or FWE for all contrasts, making the results difficult to interpret without employing each where they are best-suited.

## 3. Results

### 3.1. Behavioral results

**Figure 3:**
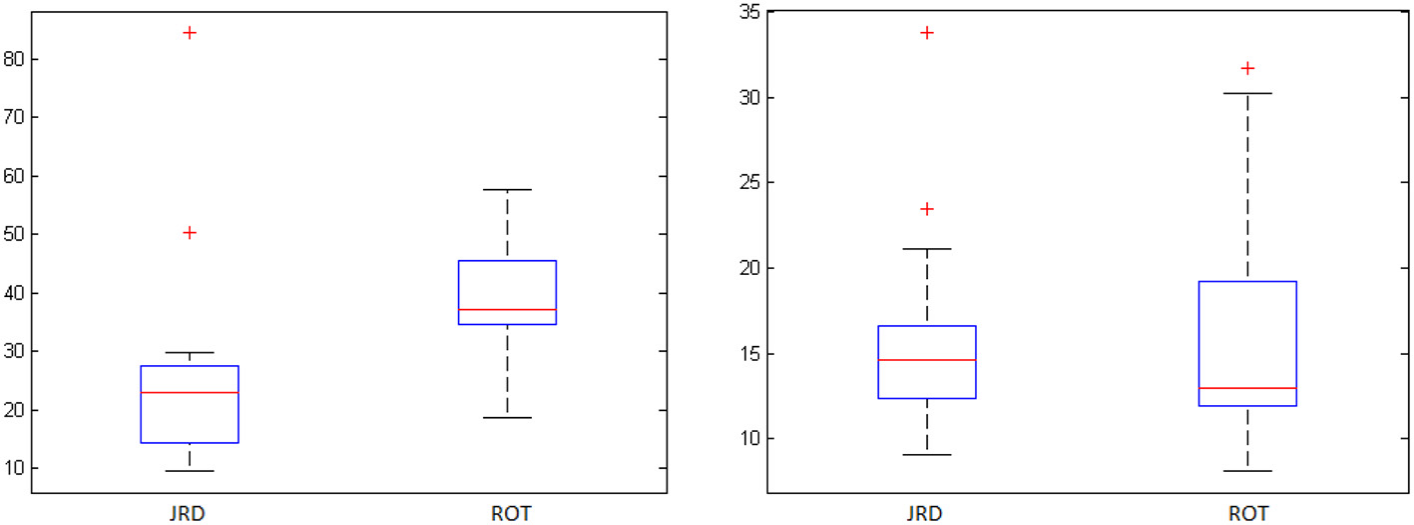
**JRD vs. ROT pointing errors (degrees) and response times (seconds) with standard deviations (‘+’ denotes an outlier)**.

To investigate the accuracy of the representations used by participants to perform the JRD and Rotation tasks, we examined participants' average absolute pointing errors from the true object location (denoted by the center of the object) and their response times from cue offset. Participants were highly accurate in both the REF and INVIS conditions, and less accurate in the Rotation task than the JRD task (see Table 1 and Figure 3). While not statistically significant due to the very high variances, there was a clear trend toward both decreased accuracy and longer response times for ROT compared to JRD trials, suggesting that participants found the ROT task to be more difficult.

### 3.2. Brain activity

Using the analytic methods described above and the parametric GLMs with pointing error as an additional regressor, we identified brain regions that were activated in proportion to performance on the different tasks performed in the scanner (except for the REF task, where the small errors and variance made the extra performance-related regressor unnecessary). Brain areas having significant task-related activations (a simple contrast with no parametric modeling) for REF vs. baseline (where participants pointed from the same viewpoint as REF but were still able to see the virtual environment, excluding the objects themselves, so that pointing was not completely from memory), and performance-related activity for the JRD task and the ROT task are given in Tables 2–4 respectively.

**Table 2:** **Activity during REF task relative to baseline (*p_FWE_* < 0.05)**.

**REGION**	**Coord. (mm)**	**Voxels**	**T-score**
**OCCIPITAL**			
Inferior occipital	LH −40 −74 −6	1050	4.27
	LH −52 −68 −6	48	3.60
**TEMPORAL**			
Superior temporal	RH 44 −38 3	103	4.29
	LH −49 −23 6	2028	4.29
Inferior temporal	RH 40 −68 3	785	4.14
	RH 38 −41 −18	280	4.79
**PARIETAL**			
Inferior parietal	RH 56 −29 18	1471	4.91
	RH 53 −41 45	212	4.05
	LH −37 −29 42	333	4.28
	LH −43 −32 30	1363	5.38
	LH −67 −29 21	50	3.84
^*^Precuneus	RH 23 −56 48	134	3.73
	RH 8 −44 45	1769	5.70
	LH −28 −47 51	49	3.89
Superior parietal	LH −28 −56 45	336	5.41
**FRONTAL**			
Precentral gyrus	RH 47 −8 45	230	4.15
	LH −13 −20 60	1724	6.14
Inferior frontal	RH 47 4 14	342	5.19

RH, right hemisphere; LH, left hemisphere;

* *Main areas of interest*.

**Table 3:** **Performance-related activity during JRD task (*p_FDR_* < 0.05)**.

**REGION**	**Coord. (mm)**	**Voxels**	**T-score**
**OCCIPITAL**			
Cuneus	RH 8 −83 3	40	4.42
Lingual gyrus	RH 17 −74 −3	26	4.12
	RH −1 −92 −9	26	4.69
	RH 26 −62 3	201	5.34
	LH −25 −77 0	29	4.52
**MEDIAL TEMPORAL**			
^*^Hippocampus	RH 29 −38 3	21	4.32
^*^Parahippocampus	RH 26 −29 −3	86	4.35
	LH −49 −29 −12	34	4.53
**BASAL GANGLIA**			
Caudate	RH 23 −44 15	253	4.76
**TEMPORAL**			
Middle temporal	RH 69 −23 −9	17	6.20
	LH −61 −32 −9	17	4.09
**PARIETAL**			
Inferior parietal	RH 29 −56 21	721	5.86
**CINGULATE CORTEX**			
Posterior cingulate	RH 11 −26 24	103	4.64
	LH −22 47 3	894	5.63
^*^Retrosplenial cortex	RH 2 −53 24	45	4.24
Anterior cingulate	LH −4 31 12	34	4.15
	LH −1 40 15	5	3.89
	LH −7 46 3	5	3.92

RH, right hemisphere; LH, left hemisphere;

* *Main areas of interest*.

**Table 4:** **Performance-related activity during the ROT task (*p_FDR_* < 0.05)**.

**Region**	**Coord. (mm)**	**Voxels**	**T-score**
**OCCIPITAL**			
Cuneus	RH 11 −98 9	474	3.80
	LH −4 −86 15	1447	3.21
Lingual gyrus	LH −1 −92 −3	35	2.62
	LH −25 −77 −6	51	2.55
Fusiform gyrus	RH 56 −14 −24	229	3.62
Middle occipital	RH 43 −86 12	90	3.27
**MEDIAL TEMPORAL**			
^*^Hippocampus	LH −31 −14 −9	112	2.95
^*^Parahippocampus	RH 25 1 −10	107	2.89
	LH −37 −41 −3	434	3.18
	LH −25 −38 3	32	2.45
	LH −31 −53 9	308	3.21
	LH −43 −8 −15	2732	5.10
Uncus	RH 26 −27 −30	34	3.00
	LH −22 1 −30	50	2.69
	LH −22 −11 −30	47	2.74
**TEMPORAL**			
Superior temporal	RH 63 −56 18	1180	3.04
	LH −28 10 −33	877	3.60
Middle temporal	RH 53 7 −21	258	4.44
Inferior temporal	LH −37 −8 −42	59	3.47
	LH −67 −59 −9	98	3.27
**PARIETAL**			
Inferior parietal	RH 50 −65 45	797	3.40
^*^Precuneus	LH −7 −67 64	21	2.52
Postcentral gyrus	RH 29 −23 39	269	3.58
**FRONTAL**			
Insular cortex	RH 29 10 −12	414	3.74
Inferior frontal	RH 53 25 3	115	3.37
	LH −37 31 −3	103	2.83
	LH −43 28 −15	645	3.66
Middle frontal	RH 50 37 −6	248	3.33
	LH −16 43 −18	235	4.47
	LH −28 10 54	132	3.09
Medial frontal	RH 11 46 15	4589	4.48
	LH −43 16 24	54	2.57
Superior frontal	LH −16 43 36	3223	4.24
	LH −13 71 −3	40	3.21
	LH −16 52 48	98	2.91
	LH −19 64 9	127	3.29
**CINGULATE CORTEX**			
^*^Cingulate/Retrosplenial	RH 16 −47 18	28995	5.07
Posterior cingulate	LH −10 −26 36	137	3.38

RH, right hemisphere; LH, left hemisphere;

* *Main areas of interest*.

**Figure 4:**
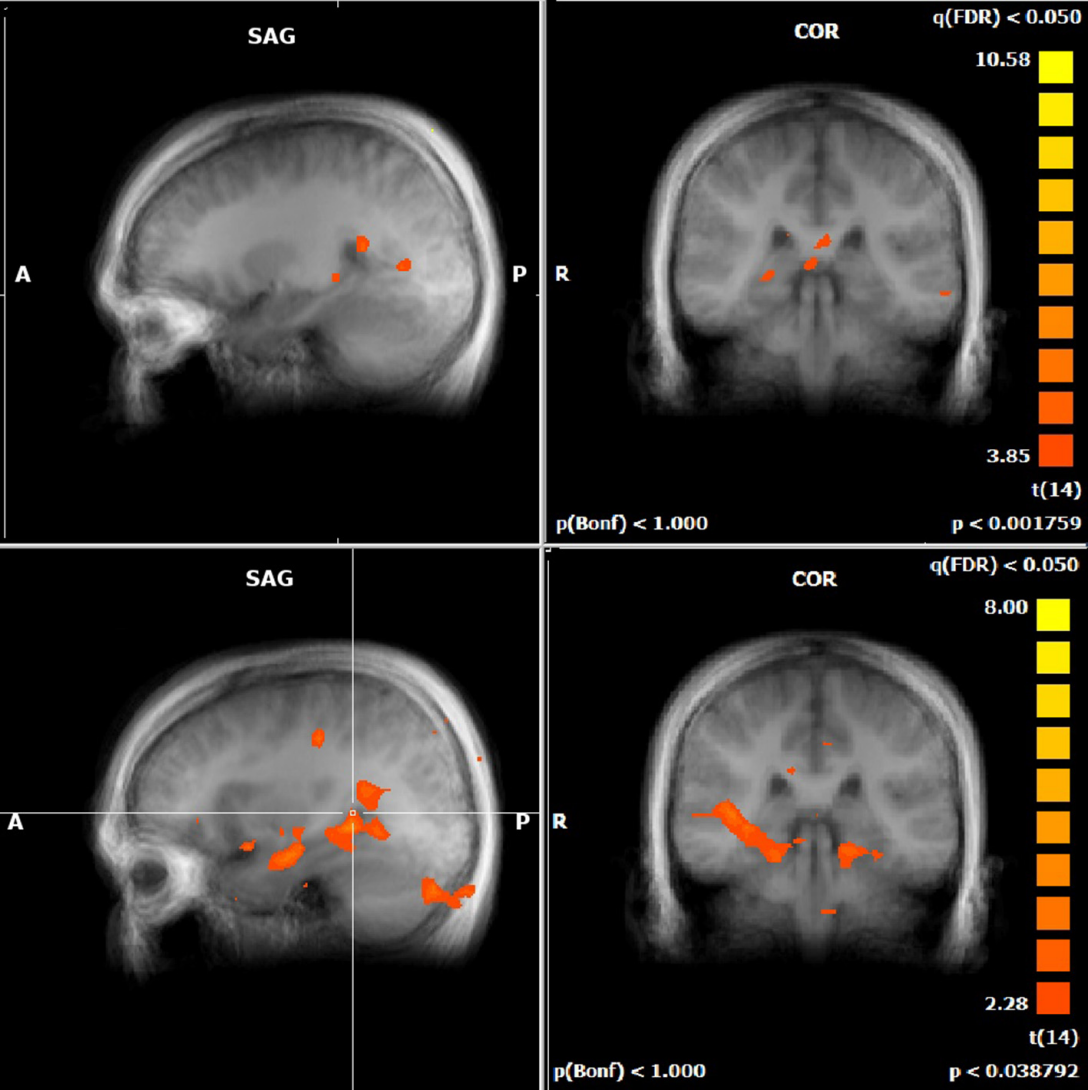
**Performance-related MTL activations for JRD (top) and ROT (bottom) conditions independently**.

For both the JRD and the ROT tasks when parameterized by performance, we found significant performance-related activations in the hippocampus (Figure 4), the parahippocampal cortex, the precuneus (however, for the JRD, precuneus activity was only seen in a non-parametric model where the pointing errors were not used as a regressor, as would be implied by the BBB model), the parietal cortex, and the retrosplenial cortex, all of which are predicted by the BBB model. In both conditions, significant task-related activations were also seen in many areas in the occipital, temporal and parietal lobes associated with visual object processing, working memory and imagery, as well as areas in the frontal lobe and cingulate cortex associated with cognitive control. Somewhat surprisingly, task-related activation was seen in the caudate nucleus in the JRD task, and in the primary somatosensory cortex in the ROT task.

**Figure 5:**
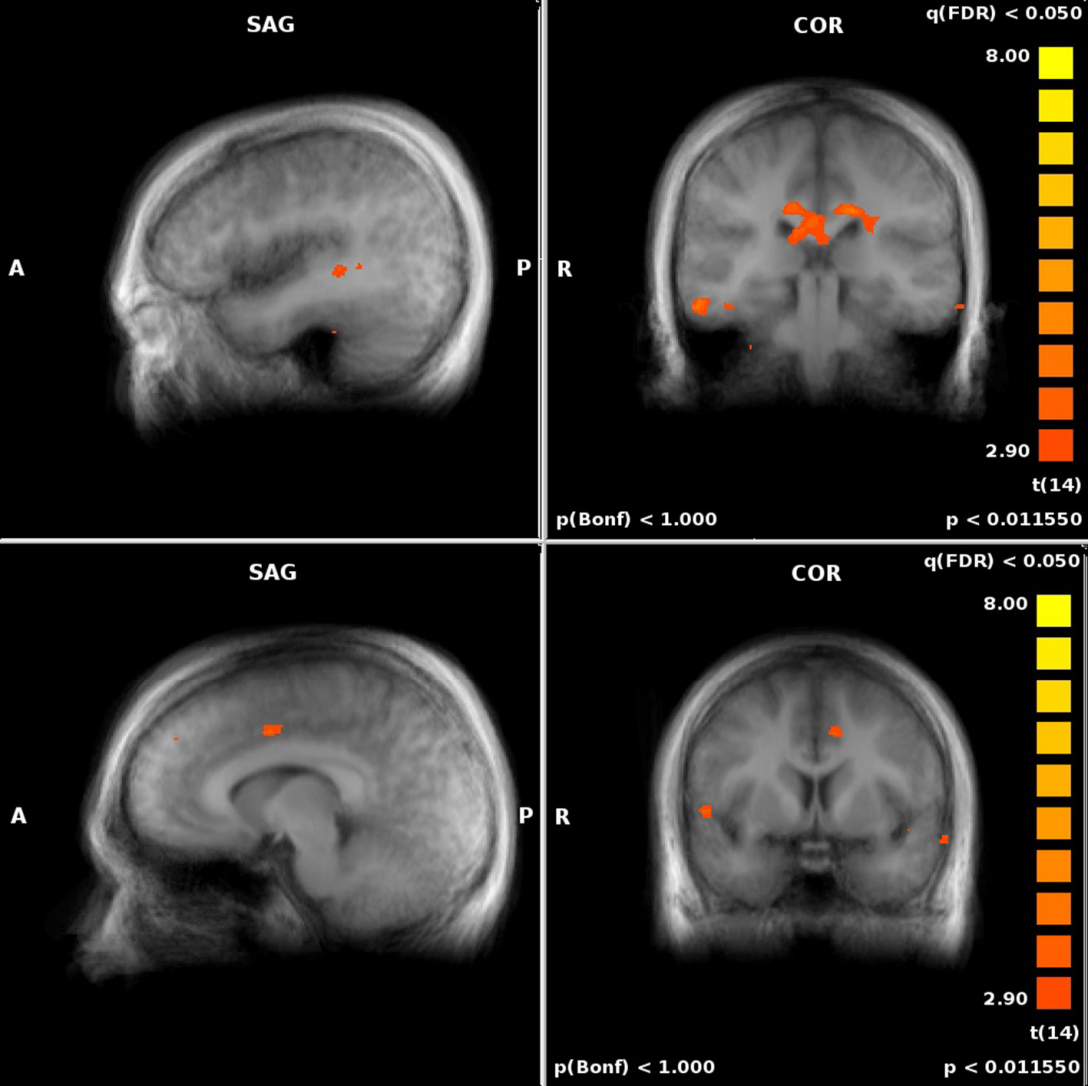
**Significant performance-related activations for JRD vs. ROT (top) and ROT vs. JRD (bottom)**.

We also assessed the difference in performance-related activation between the JRD task and the ROT task (Figure 5). When the JRD task was contrasted with the ROT task, we saw greater performance-related activation during the JRD task in the left parahippocampal gyrus, the right and left precuneus, and the right retrosplenial cortex. Additionally, the inferior temporal gyrus, middle frontal gyrus, superior frontal gyrus, middle temporal gyrus, precentral gyrus, posterior cingulate, lingual gyrus, thalamus, medial frontal gyrus, superior and inferior parietal lobules, and the middle occipital gyrus were also significantly more active during the JRD task than the ROT task (*p_FDR_* < 0.05).

There was greater activity in the ROT task contrasted with the JRD task in the superior temporal gyrus, the postcentral gyrus, the left middle temporal gyrus, the middle frontal gyrus, the medial frontal gyrus, the cuneus, and the anterior cingulate gyrus (*p_FDR_* < 0.05). Therefore, the regions identified by the BBB model were more activated by the JRD task than the ROT task.

**Figure 6:**
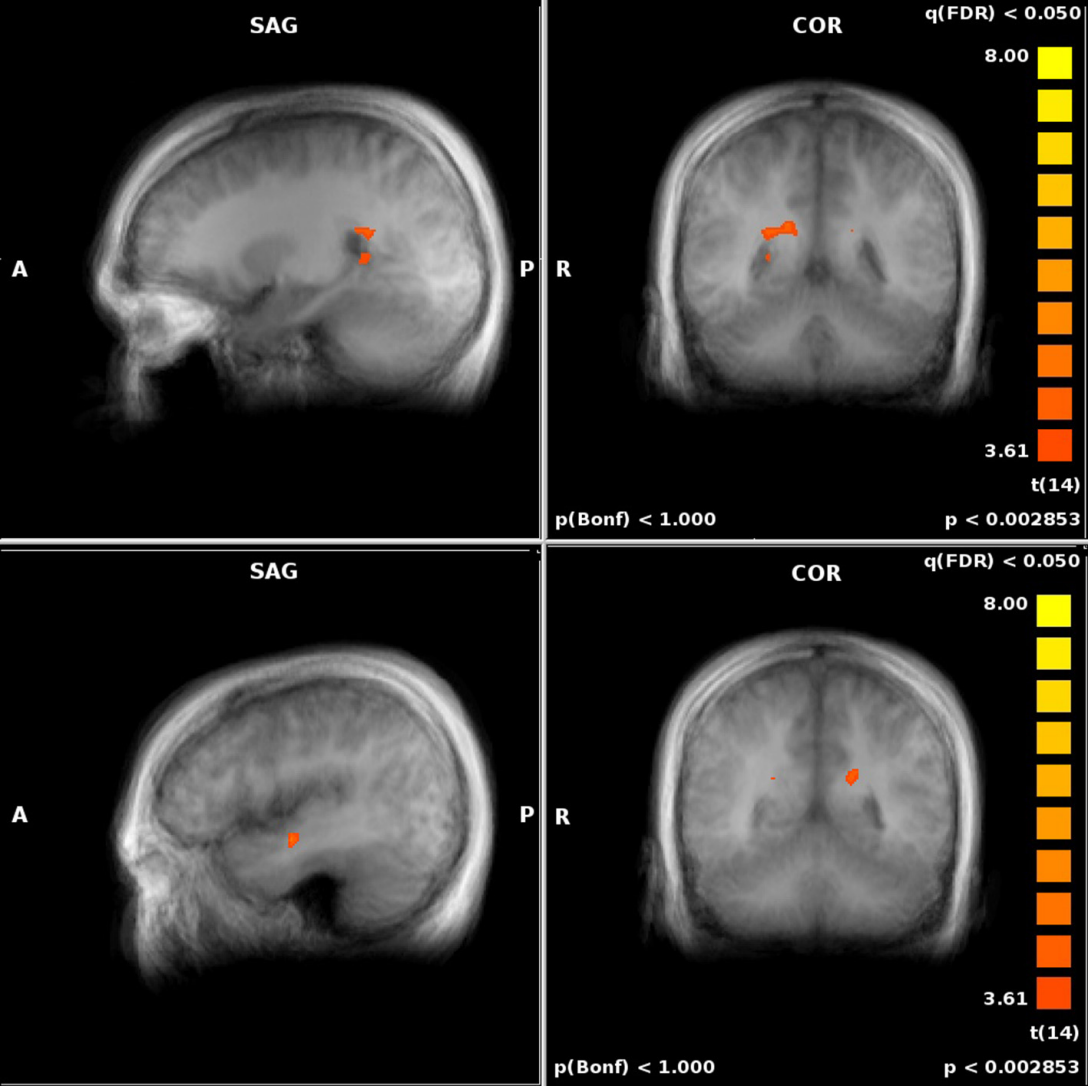
**Performance-related MTL activations for ROT vs. REF contrast**.

**Figure 7:**
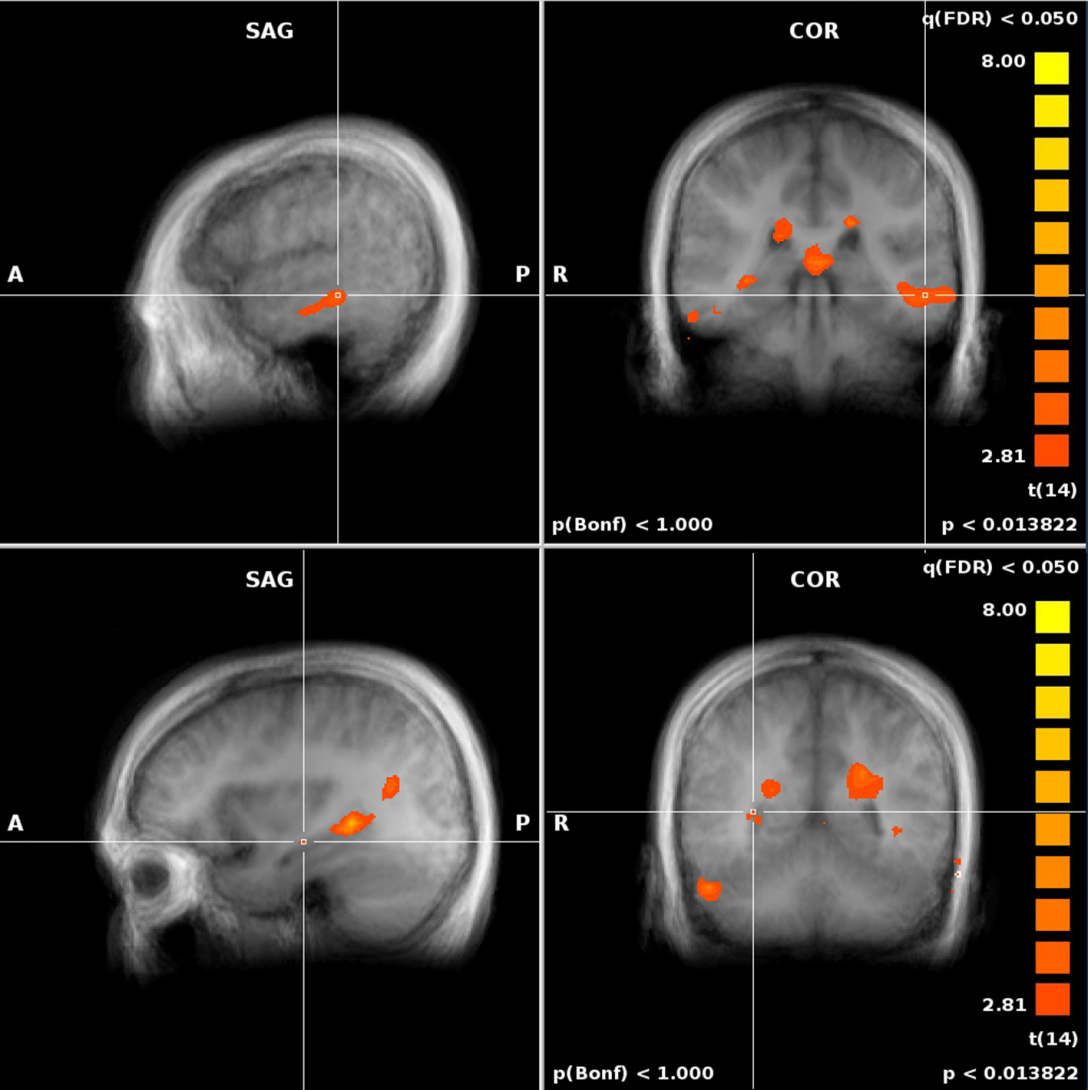
**Performance-related MTL activations for JRD vs. REF contrast**.

When comparing to REF, activity in both the JRD and the ROT tasks showed significant performance-related activation (i.e., we first subtracted the unweighted brain activity from the performance-weighted, as in our parametric model, and then subtracted the REF coefficients) in the right hippocampus, parahippocampal gyrus (left for JRD and right and left for ROT), right retrosplenial cortex, and right precuneus as predicted by the BBB model, as well as activity in the middle frontal gyrus (left and right), the anterior cingulate (left), and the superior and middle temporal gyri (left). Visual comparisons between ROT and REF and JRD and REF are shown in Figures 6, 7 respectively.

## 4. Discussion

### 4.1. The transformation circuit

The brain regions predicted by the BBB model to be involved in transforming between egocentric and allocentric representations (intraparietal sulcus/retrosplenial cortex (RSC), parahippocampal cortex and hippocampus) were active during our mental transformation tasks. Importantly, these activations correlated with task performance, consistent with our hypothesis that this neural circuit subserves the transformation between egocentric and allocentric representations. Moreover, as predicted, this circuit was more strongly activated in imagined transformations involving both rotation and translation of viewpoint (JRD) relative to transformations involving only a rotation of viewpoint (ROT). Our results in the JRD condition are consistent with those of two other recent studies that asked participants to perform spatial memory tasks after imagined viewpoint changes that included both a rotation of viewpoint and a translation of the observers location (Lambrey et al., 2012; Zhang et al., 2012). Additionally, the involvement of the hippocampus in the tasks that involve retrieval from novel viewpoints, as shown here and elsewhere (Zhang and Ekstrom, 2013), is consistent with Eichenbaum's view of the hippocampal role in relational memory (Eichenbaum and Cohen, 2001), which could be implemented by a neural circuit that supports viewpoint transformations as in the BBB model.

As expected, there were additional areas of activation in all tasks in regions associated with visuo-spatial processing and cognitive control. Unexpectedly, task-related activation of the caudate nucleus was also observed in the JRD condition. The caudate is generally associated with motor planning and goal-directed behaviors (for a review see Grahn et al., 2008). It has been implicated in prospective coding of motor responses driven by egocentric sensory and/or working memory representations (Postle and D'Esposito, 2003). This could explain the involvement of the caudate in the JRD task, if participants were planning and imagining mental navigation to the goal location in egocentric co-ordinates. Although the medial temporal lobe and basal ganglia are often portrayed as having competitive, mutually exclusive roles in spatial memory and navigation, using allocentric vs. egocentric strategies respecitvely, it is possible that a combination of the two would be employed in a complex task such as the JRD. Once an allocentrically stored spatial representation of the goal location has been retrieved, one could map this into egocentric co-ordinates to perform planning and mental navigation.

Interestingly, even though activation in the transformation circuit was more closely related to performance in the JRD condition than in the ROT condition, the ROT condition seemed to be more challenging for participants: response times were somewhat longer, and errors were somewhat higher, in the ROT than in the JRD condition (Table 1). Imagining a rotated viewpoint while holding one's location constant may pose a particular challenge to participants due to the cue conflict between one's real and imagined heading directions, especially when the avatar position was identical to the reference position in the baseline condition, which is the only position from which the environment is explicitly learned visually with feedback. This could introduce interference between the remembered locations of objects from one's current location and the target locations of objects after the imagined view rotation (May, 2004). Consistent with this sensorimotor interference interpretation, participants' response times to point to the remembered locations of objects have been found to increase in proportion to the change in imagined viewpoint, irrespective of whether there was a change in location (May, 2004). Furthermore, the ROT condition was the only one in which task-related activation was observed in the primary somatosensory cortex. Among other things, somatosensory cortex represents the position of the eye relative to the head (Wang et al., 2007) and is activated in humans when processing changes in head position (Fasold et al., 2007). Thus, activation in this region may reflect the cue conflict noted above, as participants attempt to resolve the interference between their target and actual or imagined head direction.

### 4.2. A hierarchy of allocentric representations

Much debate has been devoted to the precise role of the hippocampus in spatial memory. Is it uniquely responsible for creating allocentric spatial representations or are other areas involved? While spatial memory deficits across viewpoint changes are observed in patients with hippocampal lesions (e.g., King et al., 2002), lesions to the parahippocampal cortex and retrosplenial cortex are also associated with topographic disorientation (Habib and Sirigu, 1987; Takahashi et al., 1997). The BBB model specifies the distinct contributions of these different regions to allocentric coding: (1) posterior parietal cortex forms an egocentric representation of landmarks and boundaries, (2) posterior cingulate/retrosplenial cortex (RSC) forms a map of landmark locations modulated by egocentric or allocentric head direction respectively, depending on whether the circuit is sensory- or memory-driven, (3) parahippocampal cortex (PC) forms an allocentric map of landmark locations, and (4) hippocampal cortex cells (HC) respond to places by encoding conjunctions of landmarks, boundaries and other contextual information. Thus, damage to either the RSC, PC, or HC could cause deficits in allocentric memory and orienting.

Another controversy surrounding the role of the hippocampus in spatial coding is whether its role is time-limited, or is it always required? A challenge for the BBB model, and more generally for cognitive map theory, is to explain the finding that KC, a dense amnesic with bilateral damage to the PC and HC, was able to perform several allocentric judgments from his remote spatial memories of a familiar environment (Rosenbaum et al., 2000), including specifying alternative routes between major landmarks when the direct route was blocked. However, his remote memory for less salient landmarks was highly impaired. These findings accord with the pattern of spared and impaired spatial abilities observed in TT, a London taxi driver with bilateral hippocampal damage, who was able to navigate in a virtual model of London via major routes but was highly impaired at navigating via alternative routes (Maguire et al., 2006). The ability to orient toward salient landmarks in KC and TT could be supported by spared regions of parahippocampal and retrosplenial cortices. This would mean that the parahippocampal region, and not only the hippocampus, encodes associations amongst landmarks.

### 4.3. Limitations and future work

A limitation of the present study is that participants were drawn from a narrow demographic: male, right-handed university students with computer game experience. It was hoped that selecting for video game experience would minimize adverse reactions to immersive VR (e.g., nausea), and would keep learning time to a minimum, as gamers tend to show greater facility at traversing and learning VR layouts. The choice of right-handed males was made to minimize variability in functional activation due to sex differences and lateralization of functions. These benefits come at a cost to the generalizability of our results. One of the most well-documented sex differences is in spatial cognition (e.g., Voyer et al., 1995), a difference that often holds in VR experiments (e.g., Levin et al., 2005). However, sex differences do not manifest on all spatial tasks. For example, in a virtual 8-arm maze task, male and female participants were equally likely to report the use of spatial vs. response strategies, and both sexes performed equally well when there were multiple landmarks; only when the environment was devoid of landmarks was a male advantage evident (Andersen et al., 2012). Equally, females out-perform males in detecting changes in object locations, but this advantage is lost when the participant must move viewpoints between encoding and test (Burgess et al., 2004).

The environment used in the present study had distal landmarks clearly visible from all points in the environment that could serve as global orienting cues. However, to encourage participants to use global configural cues and form allocentric representations, there were no local landmarks intermixed with the objects within the virtual arena. The lack of local landmarks might confer a male advantage on our task. Future work is required to explore whether there are sex differences and/or individual strategy differences in this specific task, and how they may correlate with use of the transformation circuit.

Another methodological limitation involves the use of a response pointer in the fronto-parallel plane. This method of response was chosen mainly because pilot experiments showed that pointers, cursors, or sliders in plane with the arena ground were difficult and/or time-consuming for the participant, especially with the smaller screen of the scanner, which would have interfered with both task accuracy and the number of trials available for analysis. This method of response, however, could have led to some undesired brain activity, as some participants may have mentally transformed their planned pointing response into the co-ordinates of the fronto-parallel plane. We attempted to minimize this problem by asking participants to imagine pointing as if they were immersed in the environment, which may not always eliminate undesired re-transformation, if it occurs, and may introduce an imagined transformation of the pointer on the horizontal plane. Further studies involving JRD-type responses in a visual or VR paradigm must take into consideration the pros and cons of different implementations of response input, or devise a new strategy.

Screen-based testing in a visual VR paradigm also involves one other important limitation. Since participants needed to watch the screen for cues, there may be some interference in brain activations between the actual visual information being seen by the participant in the scanner and the mental imagery we are attempting to access. We attempted to minimize this by leaving the screen blank as much as possible during time-windows of interest. However, cues and the response pointer were visible during some of this time, and there is likely to be at least some brain activation due to these, since cue complexity varied between REF, ROT, and JRD (potentially contributing to some increased activation in JRD compared to ROT). While activations due to the pointer itself are likely to be mostly filtered out by averaging and parametric modeling, it is possible that how the cues were processed mentally was correlated to some degree with the accuracy of the responses, introducing some interference. However, since surveys showed that participants generally began imagining their cued position, direction, and objects while the cue was present, this interference is unavoidable with the current visual paradigm and setup.

Due to the limitation in the scanning time available for each participant to perform a sufficient number of trials, we were not able to accommodate an additional task, pointing from a fixed viewing direction with a translation only (i.e., the opposite half of the JRD that ROT encompasses). Though this task does not directly address the questions we have posed for this study, it would shed light on interesting and related questions on the differences between rotation-only and translation-only processing, and their relative contributions to the JRD task. Future work is required to tease apart the independent contributions of rotation vs. translation vs. rotation plus translation to behavioral performance in the tasks reported here, as well as the effects of rotation and translation magnitude on performance and brain activity.

We have focused in this paper on the effects of task demands such as rotation and translation on the neural circuits involved in imagined pointing responses. Other potential sources of variability in participants' responses in the ROT and JRD tasks are the degree of imagined heading disparity and object-target disparity. For example, a well established finding is that tasks requiring a rotation of imagined perspective incur a reaction time cost in proportion to the degree of heading direction disparity (e.g., Rieser, 1989). This has been taken as evidence of a mental self-rotation process that continuously updates in proportion to the degree of imagined rotation. On the other hand, others have called into question this interpretation and suggested instead that it is the interference between actual and imagined heading directions that causes the cost in reaction time (May, 2004; Wang, 2005). Our current results cannot differentiate whether the mental transformation processes studied here involve a “jump in viewpoint” that is independent of degree of rotation or a gradual viewpoint rotation that takes more time for larger angular changes. All we can conclude is that the same transformation circuit is active in both the ROT and JRD conditions in proportion to error, suggesting that the process of transforming one's viewpoint engages the same circuit irrespective of the degree of cue conflict. Future work is required to systematically manipulate heading and object direction disparity and determine whether these variables would be additional modulators of activation in the spatial transformation circuit investigated here.

### Conflict of interest statement

The authors declare that the research was conducted in the absence of any commercial or financial relationships that could be construed as a potential conflict of interest.

## References

[B1] Aguirre G. K. D'Esposito M. (1999). Topographical disorientation: a synthesis and taxonomy. Brain 122, 1613–1628 10.1093/brain/122.9.1613 10468502

[B2] Andersen N. E. Dahmani L. Konishi K. Bohbot V. D. (2012). Eye tracking, strategies and sex differences in virtual navigation. Neurobiol. Learn. Mem. 97, 81–89 10.1016/j.nlm.2011.09.007 22001012

[B3] Anderson M. I. Jeffery K. J. (2003). Heterogeneous modulation of place cell firing by changes in context. J. Neurosci. 23, 8827–8835 14523083 10.1523/JNEUROSCI.23-26-08827.2003 PMC6740394

[B4] Bohbot V. D. Kalina K. S. Spackova N. (1998). Spatial memory deficits in patients with lesions to the right hippocampus and to the right parahippocampal cortex. Neuropsychologia 36, 1217–1238 10.1016/S0028-3932(97)00161-9 9842767

[B5] Burgess N. Spiers H. J. Paleologou E. (2004). Orientational manoeuvres in the dark: dissociating allocentric and egocentric influences in spatial memory. Cognition 94, 149–166 10.1016/j.cognition.2004.01.001 15582624

[B6] Byrne P. Becker S. Burgess N. (2007). Remebering the past and imagining the future: a neural model of spatial memory and imagery. Psychol. Rev. 114, 340–375 10.1037/0033-295X.114.2.340 17500630 PMC2678675

[B7] Eichenbaum H. Cohen N. J. (2001). From Conditioning to Conscious Recollection: Memory Systems of the Brain. Oxford: Oxford University Press

[B8] Ekstrom A. Kahana M. Caplan J. Fields T. Isham E. Newman E. (2003). Cellular networks underlying human spatial navigation. Nature 425, 184–187 10.1038/nature01964 12968182

[B9] Epstein R. Kanwisher N. (1998). A cortical representation of the local visual environment. Nature 392, 598–601 10.1038/33402 9560155

[B10] Fasold O. Heinau J. Trenner M. U. Villringer A. Wenzel R. (2007). Proprioceptive head posture-related processing in human polysensory cortical areas. Neuroimage 40, 1232–1242 10.1016/j.neuroimage.2007.12.060 18296073

[B11] Forman S. D. Cohen J. D. Fitzgerald M. Eddy W. F. Mintun M. A. (1995). Improved assessment of significant activation in functional magnetic resonance imaging (fMRI). Magn. Reson. Med. 33, 636–647 10.1002/mrm.1910330508 7596267

[B12] Formisano E. Di Salle F. Goebel R. (2006). Fundamentals of data analysis methods in fMRI, in Advanced Image Processing in Magnetic Resonance Imaging, eds Landini L. Positano V. Santarelli M. F. (New York, NY: Marcel Dekker), 481–504

[B13] Genovese C. R. Lazar N. A. Nichols T. (2002). Thresholding of statistical maps in functional neuroimaging using the false discovery rate. Neuroimage 15, 870–878 10.1006/nimg.2001.1037 11906227

[B14] Goebel R. Esposito F. Formisano E. (2006). Analysis of FIAC data with BrainVoyager QX: from single-subject to cortically aligned group GLM analysis and self-organizing group ICA. Hum. Brain Mapp. 27, 392–401 10.1002/hbm.20249 16596654 PMC6871277

[B15] Grahn J. A. Parkinson J. A. Owen A. M. (2008). The cognitive functions of the caudate nucleus. Prog. Neurobiol. 86, 141–155 10.1016/j.pneurobio.2008.09.004 18824075

[B16] Habib M. Sirigu A. (1987). Pure topographic disorientation: a definition and anatomical basis. Cortex 23, 73–85 10.1016/S0010-9452(87)80020-5 3568707

[B17] King J. A. Burgess N. Hartley T. Vargha-Khadem F. O'Keefe J. (2002). Human hippocampus and viewpoint dependence in spatial memory. Hippocampus 12, 811–820 10.1002/hipo.10070 12542232

[B18] Kobayashi Y. Amaral D. (2003). Macaque monkey retrosplenial cortex: II. Cortical afferents. J. Comp. Neurol. 466, 48–79 10.1002/cne.10883 14515240

[B19] Lambrey S. Doeller C. Berthoz A. Burgess N. (2012). Imaging being somewhere else: neural asis of changing perspective in space. Cereb. Cortex 22, 166–174 10.1093/cercor/bhr101 21625010

[B20] Levin S. Mohamed F. Platek S. (2005). Common ground for spatial cognition? A behavioral and fMRI study of sex differences in mental rotation and spatial working memory. Evol. Psychol. 3, 227–254

[B21] Maguire E. A. (2001). The retrospelnial contribution to human navigation: a review of lesion and neuroimaging findings. Scand. J. Psychol 42, 225–238 10.1111/1467-9450.00233 11501737

[B22] Maguire E. A. Nannery R. Spiers H. J. (2006). Navigation around London by a taxi driver with bilateral hippocampal lesions. Brain 129, 2894–2907 10.1093/brain/awl286 17071921

[B23] May M. (2004). Imaginal perspectives in remembered environments: transformations versus interference accounts. Cogn. Psychol. 48, 163–206 10.1016/S0010-0285(03)00127-0 14732410

[B24] O'Keefe J. (1976). Place units in the hippocampus of the freely moving rat. Exp. Neurol. 51, 78–109 10.1016/0014-4886(76)90055-8 1261644

[B25] Overte Foundation (2007). Opensimulator. Available online at: http://www.opensimulator.org

[B26] Parsons T. (2004). Sex differences in mental rotation and spatial rotation in a virtual environment. Neuropsychologia 42, 555–562 10.1016/j.neuropsychologia.2003.08.014 14728927

[B27] Postle B. R. D'Esposito M. (2003). Spatial working memory activity of the caudate nucleus is sensitive to frame of reference. Cogn. Affect. Behav. Neurosci. 3, 133–144 10.3758/CABN.3.2.133 12943328

[B28] Rieser J. J. (1989). Access to knowledge of spatial structure at novel points of observation. J. Exp. Psychol. Learn. Mem. Cogn. 15, 1157–1165 10.1037/0278-7393.15.6.1157 2530309

[B29] Rosenbaum R. S. Priselac S. Kohler S. Black S. E. Gau F. Nadel L. (2000). Remote spatial memory in an amnesic person with extensive bilateral hippocampal lesions. Nat. Neurosci. 3, 1044–1048 10.1038/79867 11017178

[B30] Shelton A. L. McNamara T. P. (1997). Multiple views of spatial memory. Psychon. Bull. Rev. 4, 102–106 10.3758/BF03210780

[B31] Sherrill K. R. Erdem U. M. Ross R. S. Brown T. I. Hasselmo M. E. Stern C. E. (2013). Hippocampus and retrosplenial cortex combine path integration signals for successful navigation. J. Neurosci. 33, 19304–19313 10.1523/JNEUROSCI.1825-13.2013 24305826 PMC3850045

[B32] Sulpizio V. Committeri G. Lambrey S. Berthoz A. Galati G. (2013). Selective role of lingual / parahippocampal gyrus and retrosplenial complex in spatial memory across viewpoint changes relative to the environmental reference frame. Behav. Brain Res. 242, 62–75 10.1016/j.bbr.2012.12.031 23274842

[B33] Takahashi N. Kawamura M. Shiota J. Kasahata N. Hirayama K. Hirayama K. (1997). Pure topographic disorientation due to right retrosplenial lesion. Neurology 49, 464–469 10.1212/WNL.49.2.464 9270578

[B34] Talairach J. Tournoux P. (1988). Co-planar Stereotaxic Atlas of the Human Brain. New York, NY: Thieme

[B35] Voyer D. Voyer S. Bryden M. P. (1995). Magnitude of sex differences in spatial abilities: a meta-analysis and consideration of critical variables. Psychol. Bull. 117, 250–270 10.1037/0033-2909.117.2.250 7724690

[B36] Wang R. F. (2005). Beyond Imagination: perspective change problems revisited. Psicologica 26, 25–38

[B37] Wang X. Zhang M. Cohen I. S. Goldberg M. E. (2007). The proprioceptie representation of eye position in monkey primary somatosensory cortex. Nat. Neurosci. 10, 640–646 10.1038/nn1878 17396123

[B38] Wyss J. Groen T. V. (1992). Connections between the retrosplenial cortex and the hippocampal formation in the rat: a review. Hippocampus 2, 1–11 10.1002/hipo.450020102 1308170

[B39] Zhang H. Copara M. Ekstrom A. D. (2012). Differential recruitment of brain networks following route and cartographic map learning of spatial environments. PLOS ONE 7:e44886 10.1371/journal.pone.0044886 23028661 PMC3445610

[B40] Zhang H. Ekstrom A. (2013). Human neural systems underlying rigid and flexible forms of allocentric spatial representation. Hum. Brain Mapp. 34, 1070–1087 10.1002/hbm.21494 22786703 PMC6870227

